# The effects of two common edible herbs, *Ipomoea aquatica* and *Enhydra fluctuans*, on cadmium-induced pathophysiology: a focus on oxidative defence and anti-apoptotic mechanism

**DOI:** 10.1186/s12967-015-0598-6

**Published:** 2015-07-28

**Authors:** Tarun K Dua, Saikat Dewanjee, Ritu Khanra, Niloy Bhattacharya, Bhuvan Bhaskar, Muhammad Zia-Ul-Haq, Vincenzo De Feo

**Affiliations:** Advanced Pharmacognosy Research Laboratory, Department of Pharmaceutical Technology, Jadavpur University, Kolkata, 700032 India; The Patent Office, Karachi, 74470 Pakistan; Department of Pharmacy, University of Salerno, 84084 Fisciano, Salerno Italy

**Keywords:** Apoptosis, Cd toxicity, *Enhydra fluctuans*, *Ipomoea aquatica*, Oxidative stress

## Abstract

**Background:**

*Ipomoea aquatica* (Convolvulaceae) and *Enhydra fluctuans* (Asteraceae), two aquatic vegetables, are traditionally used against heavy metal toxicity in traditional medicines in India. The present study aimed to explore the protective role of edible (aqueous) extracts of *I. aquatica* (AEIA) and *E. fluctuans* (AEEF) against Cd-intoxication.

**Methods:**

The extracts were chemically standardized by spectroscopic and HPLC analysis. The cytoprotective roles of AEIA and AEEF were measured on mouse hepatocytes. The effect on redox status were measured after incubating the hepatocytes with CdCl_2_ (30 μM) along with AEIA or AEEF (400 μg/ml). The effects on the expressions of apoptotic signal proteins were estimated. The protective roles of AEIA or AEEF were measured by in vivo assay in mice. Haematological, serum biochemical, tissue redox status, Cd bioaccumulation and histological parameters were evaluated to estimate the protective role of AEIA or AEEF (100 mg/kg) against CdCl_2_ (4 mg/kg) intoxication.

**Results:**

Phytochemical analysis revealed presence of substantial quantities of phenolics, flavonoids, saponins, carbohydrates and ascorbic acid in AEIA or AEEF. CdCl_2_ treated murine hepatocytes showed a gradual reduction of cell viability in a concentration dependent manner with an IC_50_ of ~30 μM. CdCl_2_ treated hepatocytes exhibited significantly enhanced levels (p < 0.01) of ROS production, lipid peroxidation, protein carbonylation and NADPH oxidase with concomitant depletion (p < 0.01) of antioxidant enzymes and GSH. However, AEIA or AEEF treatment along with CdCl_2_ significantly restored the aforementioned parameters in murine hepatocytes near to normalcy. Besides, AEIA or AEEF significantly counteracted (p < 0.05–0.01) with ROS mediated alteration of transcription levels of signal proteins viz. Bcl-2, BAD, Cyt-C, Caspases, Fas and Bid. In in vivo bioassay, CdCl_2_ treatment caused significantly high Cd bioaccumulation and oxidative stress in the liver, kidney, heart, brain and testes in mice. In addition, the haematological and serum biochemical parameters were significantly altered in the CdCl_2_ treated animals. Simultaneous administration of AEIA or AEEF could significantly restore the tested parameters to the near-normal status.

**Conclusion:**

The extracts would offer the overall protective effect via counteracting with Cd mediated oxidative stress and/or promoting the elimination of Cd by chelating.

## Background

Cadmium (Cd), a toxic heavy metal, exerts deleterious effect to the majority of human organs. With increasing industrial and anthropogenic activities, over past few decades, emanations of Cd into the environment has increased significantly than normal limits [1]. Therefore, the risk of Cd exposure is constantly increasing. Significantly high half-life, faster absorption and low rate of excretion promote the bioaccumulation of Cd above toxic level within a short span of time [2, 3]. In chronic exposure, Cd has been considered to be a multi-target toxicant, and it causes damage of vital organs namely liver, kidney, heart, brain and testes [4]. The exact mechanism of Cd toxicity is not quite clear, however, Cd mediated free radical generation is proposed to be the principle mechanism of Cd pathogenesis [5]. The reactive oxygen species (ROS) directly react with cellular macromolecules mainly lipids, proteins and nucleic acids and that can ultimately lead to cell death [5, 6]. Besides, ROS activates NADPH oxidase [7] and mitochondrial dysfunction, both participate in the apoptotic events within the tissues [8]. Considering the mechanisms of Cd toxicity, it would be important to consider antioxidants to counteract with these toxic manifestations. Initially, various types of metal chelating agents and antagonists have been exploited to counteract with Cd toxicity, however, several undesirable side effects and contraindications have largely restricted their applications [9]. On other hand, many of edible plants are known to possess significant antioxidant activities, as they are rich in phyto-antioxidants [10]. Therefore, dietary antioxidants would offer a protective role against Cd toxicity. Because of easy availability, low cost and no side effect, dietary antioxidants would have huge scope to serve as a therapy of Cd-intoxication.

*Ipomoea aquatica* Forssk. (Convolvulaceae) and *Enhydra fluctuans* Lour. (Asteraceae) are two aquatic vegetables distributed in Southern Asia, India, and China. These are commonly consumed as green leafy vegetables and they have high nutritive values to human beings. *I. aquatica* has been used in folk medicine against different diseases including diabetes [11], hypertension [12], liver malfunction [13], constipation [14] and in the treatment of opium and arsenic poisoning [15]. On other hand, *E. fluctuans* possesses ethnomedicinal significance as anti-inflammatory [16], anthelmintic [16], analgesic [17], hepatoprotective [18] and hypotensive [18] agent. A Literature survey revealed presence of flavonoids, phenolic compounds, β-carotene and ascorbic acid in both the plants [18, 19]. Considering the presence of phyto-antioxidant molecules, the present study was designed to evaluate the possible protective role of aqueous extracts of the two aforementioned edible plants in Cd-toxicity. A suitable in vitro model (on isolated murine hepatocytes) was used to study the effect of the extracts against Cd-toxicity on isolated cells. Cell viability was measured to assess the cyto-protective role of the extracts. The effects of test materials on markers exhibiting cellular redox status were estimated. In addition, the expressions of redox sensitive transcription proteins were examined in mouse hepatocytes in absence and presence of test extracts. Based on in vitro results, we have tested the test materials in vivo. Haematological, biochemical and redox markers were estimated in vivo in experimental mice. Finally, histological studies were performed to show the overall protective role of *I. aquatica* and *E. fluctuans* against Cd-induced toxic manifestations.

## Methods

### Chemicals

The 1-chloro-2,4-dinitrobenzene, ammonium sulphate, 2,4-dinitrophenylhydrazine, ethylene diaminetetraacetic acid, 5,5-dithiobis(2-nitrobenzoic acid), *N*-ethylmaleimide, nitroblue tetrazolium, reduced nicotinamide adenine dinucleotide, potassium dihydrogen phosphate, phenazinemethosulphate, sodium pyrophosphate, reduced glutathione, sodium azide, thiobarbituric acid, 5-thio-2-nitrobenzoic acid and trichloro acetic acid were purchased from Sisco Research Laboratory, Mumbai, India. Bradford reagent, bovine serum albumin and cadmium chloride were procured from Sigma-Aldrich Chemical Company, St. Louis, USA. All antibodies for immunoblotting were purchased from Sigma-Aldrich Chemical Company St. Louis, USA. HPLC grade solvents (acetic acid, formic acid, methanol and acetonitrile) were procured from Merck, Mumbai, India.

### Preparation of extract

Aerial parts of *I. aquatica* and *E. fluctuans* were collected in the month of September, 2012 from West Midnapore, India. The plants were authenticated by the Taxonomists, Botanical Survey of India, Howrah, India. The voucher specimens JU/PT/PC/07/12 and JU/PT/PC/09/12 have been deposited at Advanced Pharmacognosy Research Laboratory. The plants were dried in an incubator (40 ± 5°C, for 72 h) and pulverized into fine powder. The powdered plant materials were macerated with double distilled water containing 1% chloroform (v/v) for 48 h at 30 ± 5°C with continuous stirring. Addition of 1% chloroform ensures protection against microbial growth within the aqueous system without affecting polarity. The extracts were filtered to remove particulate matters and lyophilized (Heto FD 3 Drywinner, Allerod, Denmark) to yield the powdered crude extracts AEIA (~11.7%, w/w) and AEEF (~12.5%, w/w). The testing samples were prepared by dissolving lyophilized powder in distilled water containing 1% tween 80 (v/v) prior to in vivo experiment, whereas for in vitro assay, the extracts were solubilized in DMSO and diluted with autoclaved water into desired concentration (resultant ≤0.4% DMSO in contact to cells to avoid DMSO induced cytotoxicity).

### Phytochemical analysis

Quantitative analysis for phytochemicals present within the extracts (AEIA and AEEF) was determined spectrophotometrically by evaluating total flavonoids [21], total phenolics [22], total saponins [23] and total carbohydrates [24] content. The identification of individual phenolic and flavonoid compounds was done in Dionex Ultimate 3000 HPLC system (Dionex, Germany), using a reverse phase C-18 column (250 × 4.6 mm, particle size 5µ) and UV detector. Methanol (HPLC grade) was used to prepare samples which were later filtered by cellulose nylon membrane filter (0.45 µm). The aliquots of the filtrate were eluted with an isocratic solvent mixture comprising methanol: acetonitrile: acetic acid: *o*-phosphoric acid: water (20:10:1:1:20) for flavonoids, methanol: water: acetic acid (75:24:1) for phenolic compounds and 25 mM phosphate buffer (pH 3.0 with phosphoric acid) for ascorbic acid with the flow rate of 1 ml/min and detected at 352, 254 and 230 nm, respectively. The quantity of ascorbic acid within the extracts was estimated by HPLC analysis.

### Animals

Healthy Swiss albino mice (♂) of age between 1 and 2 months, weighing between 20 and 30 g were employed in this study. Animals were maintained under standard laboratory conditions of temperature (25 ± 2°C), relative humidity (50 ± 15%), 12 h light–dark cycle, standard diet and water ad libitum. The principles of Laboratory Animals care [25] and the instructions given by our institutional animal ethical committee (Reg no 0367/01/C/CPCSEA) were followed throughout the experiment.

### In vitro bio-assay

#### Hepatocyte isolation

The mice were subjected to CO_2_ euthanasia and sacrificed by cervical dislocation. The livers were excised, cleaned and washed with phosphate buffer saline (pH 7.4). Hepatocytes were isolated from mouse liver by the method of Sarkar and co-workers [26] with little modifications. Briefly, hepatocytes were isolated by collagenase perfusion followed by mechanical disruption. Hepatocytes (~90% viability) were passed through a wide bore syringe, filtered and centrifuged (500 rpm) for 5 min. The pellet was resuspended in Dulbecco’s Modified Eagle Medium containing 10% Fetal bovine serum and incubated at 37°C and 5% CO_2_ tension to achieve monolayer in the culture flask.

#### Determination of cytotoxic effect of CdCl_2_

Concentration dependent cytotoxic effect of CdCl_2_ was determined by cell viability assay. Briefly, different sets of hepatocytes (~2 × 10^6^ cells/set) were incubated with CdCl_2_ (0.25–10,000 µM) at 37°C and 5% CO_2_ tension for 2 h. The percents of viable cells were counted using MTT (3-(4, 5-dimethylthiazolyl-2)-2, 5-diphenyltetrazolium bromide) assay [27].

#### Assessment of cytoprotective role of AEIA and AEEF

A cell viability assay has been performed to measure cytoprotective role of extracts (AEIA and AEEF) against Cd-induced cytotoxicity. Briefly, different sets of hepatocytes (~2 × 10^6^ cells/set) were incubated at 37°C with CdCl_2_ (30 µM) and CdCl_2_ (30 µM) along with the extracts (50, 100, 200 and 400 µg/ml) for different times (0.5 h, 1 h, 1.5 h, 2 h, 2.5 h and 3 h). The concentration of CdCl_2_ was chosen on the basis of IC_50_ value (~30 µM) of CdCl_2_ in isolated murine hepatocytes. The cell viability was determined as by the MTT assay [27]. One set without CdCl_2_ was kept as normal control. Two sets of cells treated with highest concentration of either of test extracts (400 µg/ml) (without CdCl_2_) were kept to ensure that the test extracts are non-toxic to isolated murine hepatocytes.

#### Flow cytometric analysis

The flow cytometric analysis has been performed to determine the nature of death. Briefly, CdCl_2_ at a dose of 30 µM (in presence or absence of AEIA or AEEF at a dose of 400 µg/ml) were incubated for 3 h at 37°C. The doses of Cd and extracts were selected on the basis of the observation of cell viability assay. One set without CdCl_2_ was kept as normal control. The hepatocytes were treated with propidium iodide (PI) and FITC-labelled Annexin V for 30 min at 37°C. After washing of excess of PI and Annexin V; cells were fixed and analysed by flow cytometry using FACS Calibur (Becton–Dickinson, Mountain View, USA) equipped with 488 nm argon laser light source; 515 nm band pass filter for FITC-fluorescence and 623 nm band pass filter for PI-fluorescence using Cell Quest software. A scatter plot of PI-fluorescence vs FITC-fluorescence has been prepared.

#### Hoechst staining

Hoechst staining was performed to detect cell viability by staining nucleus under fluorescence microscopy. Briefly, 2,000 cells/well were cultured in tissue culture plate and incubated at 37°C and 5% CO_2_. 24 h after seeding, the cells were incubated with CdCl_2_ + AEIA or AEEF. After 2 h, the cells were fixed in 4% paraformaldehyde in PBS for 25 min. Fixed cells were incubated with Hoechst 33258 (5 μg/ml in PBS) for 15 min. Fluorescent nuclei were scored as an index of cell viability.

#### Assays of antioxidant markers

Different sets of hepatocytes, each containing 1 ml of suspension (~2 × 10^6^ cells/ml) were used in the experiments. The protective role of AEIA or AEEF in CdCl_2_-intoxication was analyzed by incubating hepatocytes with extracts and CdCl_2_ together for 2 h at 37°C [2]. Hepatocytes were incubated for 2 h with CdCl_2_ to act as toxic control. One set without any treatment was kept as control. Generation of intracellular ROS was quantified by a previously reported method of LeBel and Bondy [28] with little modification introduced by Kim and co-workers [29] and 2′,7′-dichlorofluorescein-formation was measured by using a fluorescence spectrometer (HITACHI, Model No. F4500, Japan) at the excitation and emission wavelengths of 488 nm and 510 nm, respectively. NADPH oxidase activity was analyzed by the method described previously [30]. The extent of lipid peroxidation i.e. thiobarbituric acid reactive substances (TBARS) were measured by the method described by Ohkawa et al. [31]. The protein carbonylation was determined according to the method of Uchida and Stadtman [32]. The antioxidant enzymes superoxide dismutase (SOD), catalase (CAT), glutathione-S-transferase (GST), glutathione peroxidase (GPx), glutathione reductase (GR) and glutathione-6-phosphate dehydrogenase (G6PD) activity was measured according to the method described by Ghosh and co-workers [20]. Non-enzymatic antioxidant, reduced glutathione (GSH), level was assayed by the method of Hissin and Hilf [33].

#### Immunoblotting of signaling proteins

Samples containing proteins (20 µg) were subjected to SDS-PAGE (12%) and transferred to a nitrocellulose membrane using standard dry transfer method. Membranes were blocked (4°C;1 h) in blocking buffer containing non-fat dry milk (5%) to prevent non-specific binding and were washed with tris-buffered saline (TBS) with 0.1% Tween 20 (pH 7.6) with gentle shaking. Then the membranes were incubated with primary antibodies viz. anti-caspase 3 (1:1,000 dilution), anti-caspase 9 (1:1,000), anti-caspase 8 (1:1,000), anti-P65 NF-κB (1:1,000), anti-JNK (1: 1,000), anti-phospho-p38 (1: 1,000), anti-Bad (1:2,000), anti-Bcl-2 (1:2,000 dilution), anti-Fas (1: 1,000), anti-Bid (1: 1,000), anti-cyt C (1: 1,000) at 4°C overnight. The membranes were washed with Tris-buffered saline (TBS) with 0.1% Tween 20 (pH 7.6) with gentle shaking, incubated with appropriate HRP conjugated secondary antibody (1:3,000 dilution) for 1 h at room temperature and developed by the HRP substrate 3, 3′-diaminobenzidine tetrahydrochloride system (Bangalore Genei, India).

### In vivo bioassay

#### Experimental design

**Fig. 1:**
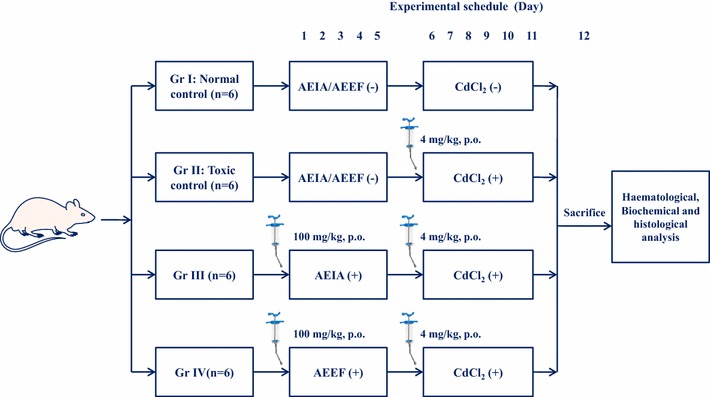
Schematic presentation of in vivo experimental protocol. ‘−’ means untreated, ‘+’ means treated.

Twenty four Swiss albino mice were divided into four groups (n = 6) and they were treated as follows:

Group I: Normal control (animals received only double distilled water as vehicle);

Group II: Toxic control (animals received CdCl_2_ through double distilled water at a dose of 4 mg/kg body weight, p.o. for 6 days, once daily) [5];

Group III: Animals were treated with AEIA (100 mg/kg body weight, p.o.) for 5 days followed by CdCl_2_ (4 mg/kg body weight, p.o., once daily) intoxication for next 6 days [5];

Group IV: Animals were treated with AEEF (100 mg/kg body weight, p.o.) for 5 days followed by CdCl_2_ (4 mg/kg body weight, p.o., once daily) intoxication for next 6 days [5];

The in vivo experimental schema has been depicted in Fig. 1. The selection of doses was based on the observation of in vitro assay and the preliminary assay conducted in our laboratory. In this manuscript, we mentioned only optimum doses of extracts to show their comparative effectiveness. The dose of CdCl_2_ was chosen on the basis of acute toxicity study in experimental mice. The LD_50_ value was found to be ~4 mg/kg. After 11 days, the animals were subjected to CO_2_ euthanasia and sacrificed by cervical dislocation. Before sacrificing the animals, blood samples were collected from retro-orbital venous plexus in Eppendorf tubes rinsed with anticoagulant for haematological assays. The organs (Liver, kidney, brain, heart and testes) were excised, cleaned and washed with phosphate buffer saline (pH 7.4). The organs were homogenized in 0.1 M Tris–HCl-0.001 M EDTA buffer (pH 7.4) and centrifuged at 12,000 g for 30 min at 4°C. The supernatant were collected and used for assaying biochemical parameters.

#### Haematological parameters

The total erythrocytes count and haemoglobin content were determined by using standard laboratory procedures. Serum biochemical parameters viz. alanine aminotransferase (ALT) and aspartate aminotransferase (AST), urea, cholesterol and triglycerides were estimated by standard kits (Span Diagnostic Limited, India).

#### Assessment of antioxidant markers related to organ dysfunction

Distribution of Cd in tissues has been measured by flame atomic absorption spectroscopy. The intercellular ROS, NADPH, TBARS level, antioxidant enzymes, non-enzymatic antioxidant were assayed following standard assay protocols mentioned earlier. Co-enzymes Q (Q_9_ and Q_10_) were isolated and estimated according to the method of Zhang and co-workers [34].

#### Histological studies

The organs from the normal and experimental mice were fixed in 10% buffered formalin and were processed for paraffin sectioning. Sections of about 5 μm thickness were stained with hematoxylin and eosin to study the histology of organs of all experimental animals [35].

### Statistical analysis

Data were statistically examined by one way ANOVA and reported as mean ± SE followed by Dunnett’s *t* test using GraphPad InStat software, version 3.05, U.S.A. The values were considered significant when *p* < 0.05.

## Results

### Phytochemical analysis

**Fig. 2:**
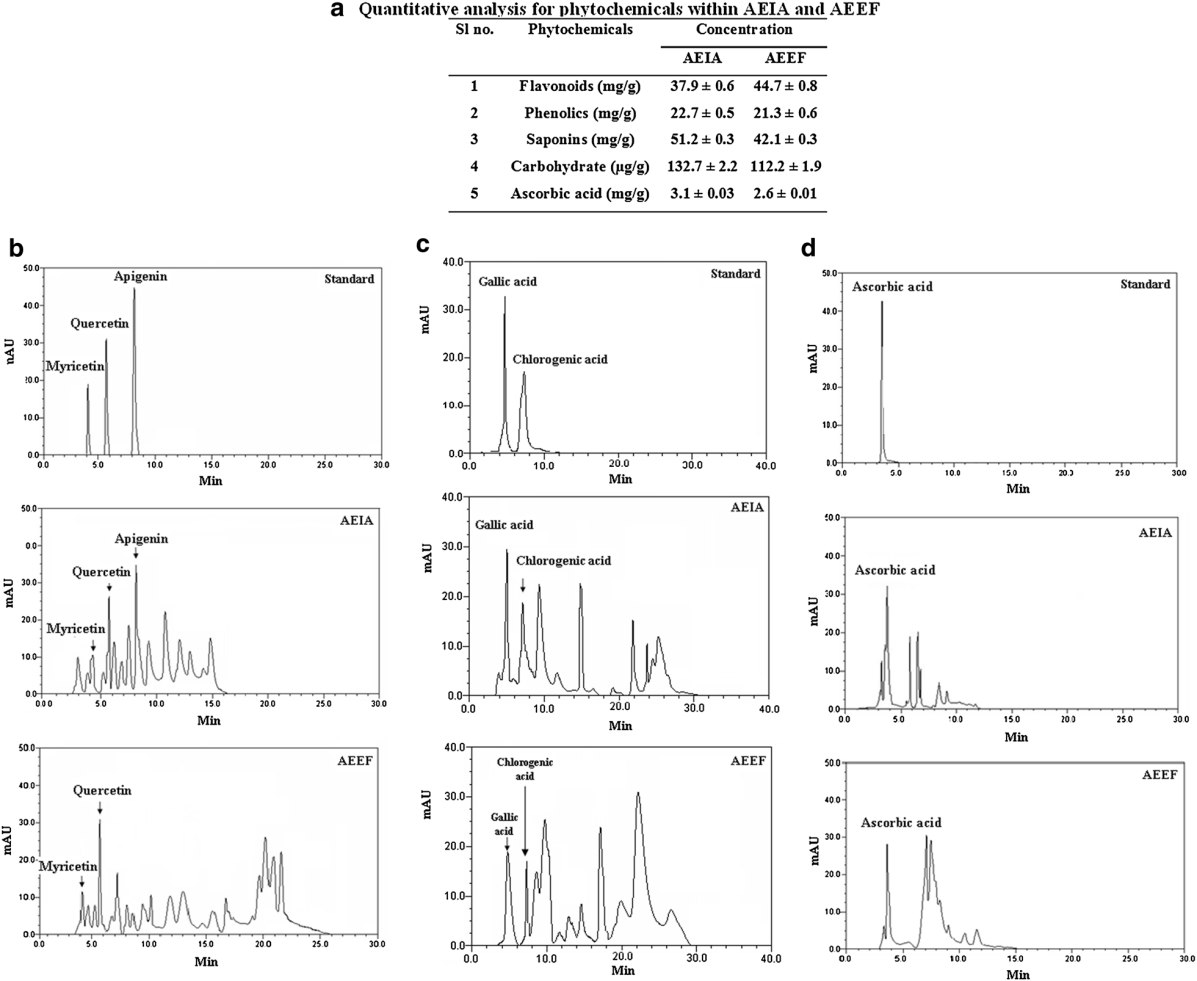
Phytochemical analysis of AEIA and AEEF. **a** Quantitative analysis of AEIA and AEEF. Values are expressed as mean ± SE. **b** Representative HPLC chromatograph of standard flavonoids viz. myricetin (R_t_: 4.08) quercetin (R_t_: 5.70) and apigenin (R_t_: 8.16) and flavonoids in AEIA and AEEF. **c** Representative HPLC chromatograph of standard phenolic compounds viz. gallic acid (R_t_: 4.03) and chlorogenic acid (R_t_: 7.24) and phenolic compounds in AEIA and AEEF. **d** Representative HPLC chromatograph of standard ascorbic acid (R_t_: 3.91) and ascorbic acid in AEIA and AEEF.

Quantities of phytochemicals were depicted in Fig. 2a.The identification of phenolic compounds and flavonoids were done by RP-HPLC analysis and comparing retention time (R_t_) and UV spectra with standard flavonoids and phenolic compounds. HPLC analysis revealed presence of flavonoids namely myricetin, quercetin and apigenin in AEIA, while, HPLC analysis reported presence of myricetin and quercetin in AEEF (Fig. 2b). In search of phenolic compounds, both the extracts showed presence of gallic acid and chlorogenic acid (Fig. 2c). Significant quantities of ascorbic acid in AEIA and AEEF (Fig. 2d) were also detected.

### Effect of AEIA and AEEF in Cd toxicity in isolated murine hepatocytes

#### Effect on Cd induced cytotoxicity

**Fig. 3:**
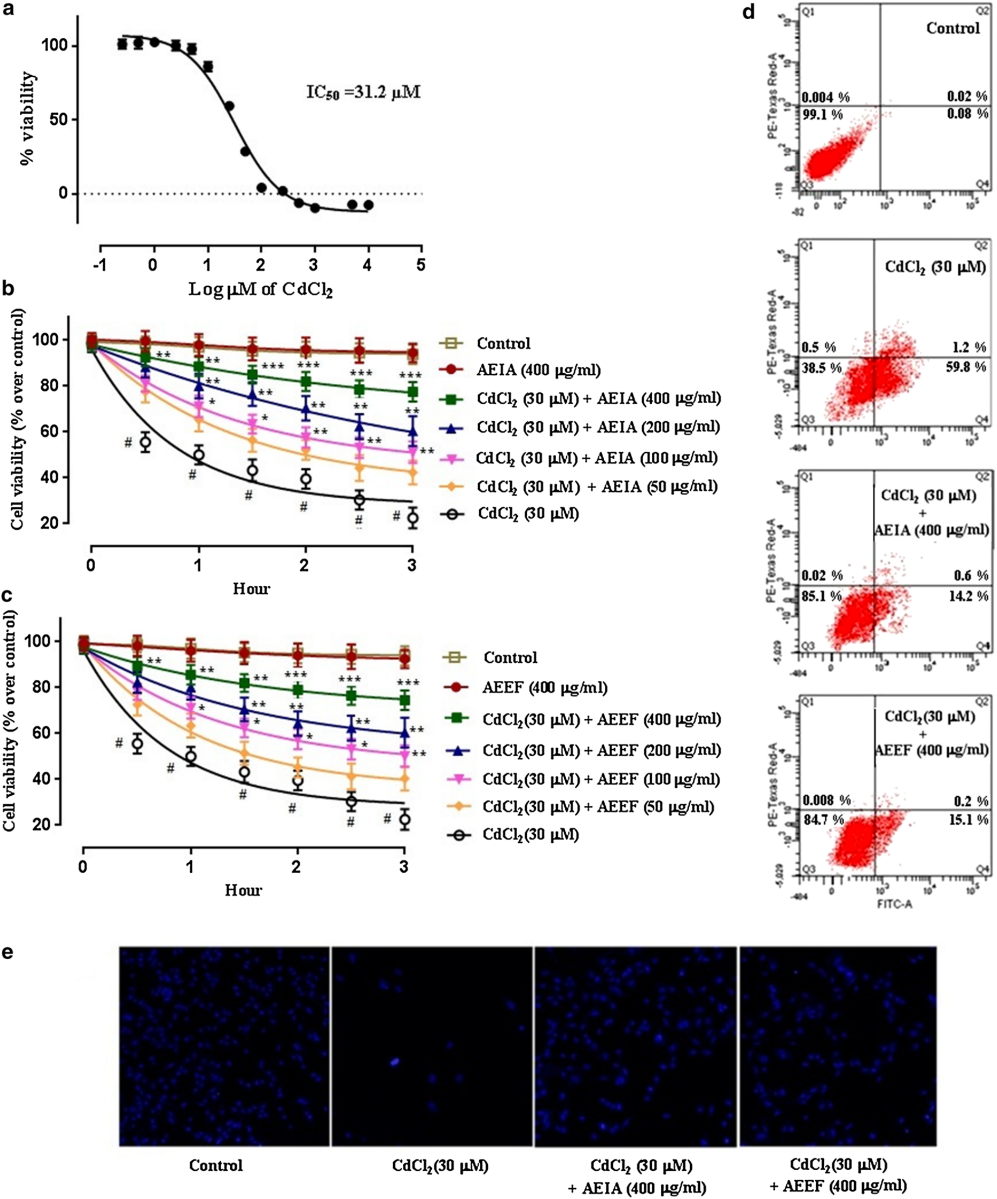
Cell viability studies in absence (CdCl_2_) and presence of AEIA OR AEEF in vitro. **a** Effect of CdCl_2_ at different concentrations in cell viability in isolated mouse hepatocytes. **b** Time and dose-dependent effect on cell viability in absence (CdCl_2_) and presence of AEIA (A) in isolated murine hepatocytes. **c** Time and dose-dependent effect on cell viability in absence (CdCl_2_) and presence of AEEF (**a**) in isolated murine hepatocytes.Values are expressed as mean ± SE (n = 3). ^#^Values differ significantly (p < 0.01) from normal control. *Values differ significantly (p < 0.05) from toxic control. **Values differs significantly (p < 0.01) from toxic control. **Values differ significantly (p < 0.001) from toxic control. **d** Percentage distribution of apoptotic and necrotic cells in absence (CdCl_2_) and presence of AEIA or AEEF by flow cytometric analysis. **e** Hoechst staining of murine hepatocytes in absence (CdCl_2_) and presence of AEIA and AEEF.

Cell viability is an important indicator of finding the degree of cytotoxicity, caused by any toxicant. Figure 3a depicts the dose dependent effect of CdCl_2_ in isolated murine hepatocytes. Hepatocytes incubated with CdCl_2_ (0.25–10,000 μM) for 2 h exhibited the reduction in cell viability in a concentration dependant manner. The IC_50_ value was found to be 31.2 μM (~30 μM). Figure 3b, c depicted the protective role of AEIA and AEEF in CdCl_2_ induced cytotoxicity in isolated murine hepatocytes, respectively. The simultaneous treatment of hepatocytes with AEIA and AEEF (50, 100, 200 and 400 µg/ml) (30 µM) prevented the CdCl_2_ mediated reduction in cell viability up to 3 h in a concentration dependent manner. The best cytoprotective role was found at the dose of 400 µg/ml in either of extract. AEIA (400 µg/ml) and AEEF (400 µg/ml) could restore cell viability of ~77.5 and 74.3% at 3 h, respectively. However, the cells treated with CdCl_2_ (30 µM) alone exhibited gradual reduction of cell viability up to 3 h. On other hand, the untreated murine hepatocytes (control) were kept as control and linearly restore cell viability throughout the course of study (~98%). The cells treated with the individual extract (without CdCl_2_) maintained almost similar pattern of viability up to 3 h as compared with control group.

To investigate the nature of cell death, hepatocytes were assessed by flow cytometric analysis. Flow cytometric data (Fig. 3d) revealed that, in comparison to control, Cd-intoxicated hepatocytes showed maximum Annexin V-FITC-binding (~38.5%), but very little PI staining (~1.2%) indicating majority of cells were apoptotic. In AEIA and AEEF treated hepatocytes, the number of apoptotic cells was significantly low (~14.2 and 15.1%, respectively) indicating a possible cytoprotective role of AEIA and AEEF from Cd-induced apoptosis. While, control group showed very little apoptotic (~0.08%) cells as compared with viable cells (~99.1%).

The cytoprotective effect of AEIA and AEEF was confirmed by fluorescence microcopy after Hoechst staining (Fig. 3e) of hepatocytes under different treatments. CdCl_2_ treated hepatocytes exhibited significantly less number of visible nuclei, however, the visible nuclei exhibited distinct pattern of morphological changes as compared with normal hepatocytes. The simultaneous incubation of hepatocytes with AEIA or AEEF along with CdCl_2_ significantly counteracted with cytotoxic effect of CdCl_2_ confirmed by nuclear scoring and morphology.

#### Effect on CdCl_2_ induced alteration of antioxidant markers in isolated murine hepatocytes

**Fig. 4:**
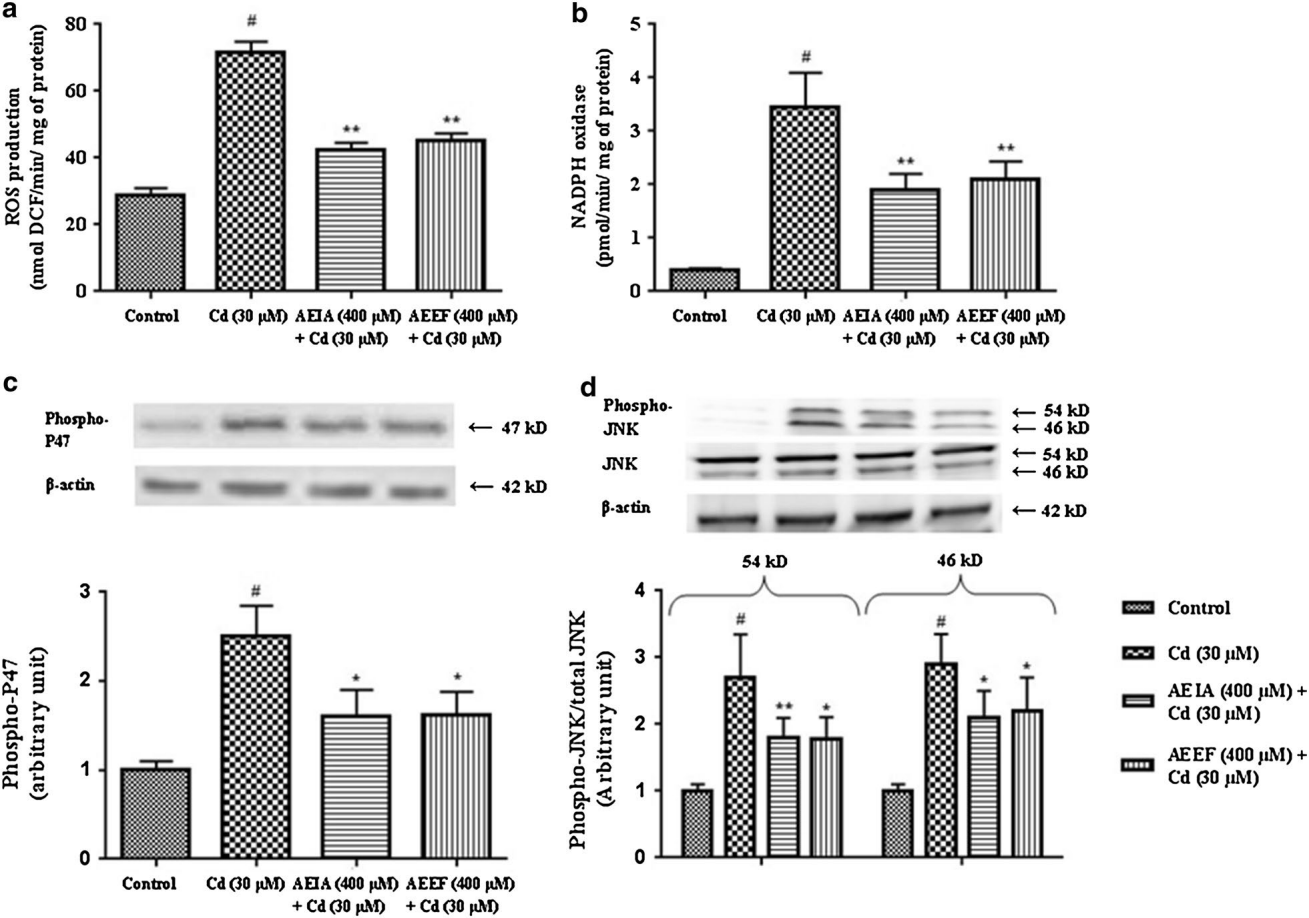
Effect on ROS production, NADPH oxidase system, phospho-P47 and JNK status in absence (CdCl_2_) and presence of AEIA and AEEF in vitro. Effect on intercellular ROS production (**a**). Effect on NADPH oxidase system (**b**). Respective western blot analysis of phospho-P47 (**c**) and phosphorylated-JNK and total JNK (**d**) followed by densitometric analysis of the respective protein levels (*bottom*) and the normal control band was given an arbitrary value of 1. β-actin was used as a loading protein. Values are expressed as mean ± SE (n = 3). ^#^Values differ significantly (p < 0.01) from normal control. *Values differ significantly (p < 0.05) from toxic control. **Values differ significantly (p < 0.01) from toxic control.

Table 1 represents the effects of AEIA and AEEF against CdCl_2_ induced alteration of antioxidant markers in isolated mouse hepatocytes. The extent of lipid peroxidation (TBARS) and protein carbonylation was significantly enhanced (p < 0.01) in Cd-intoxicated hepatocytes. The extent of lipid peroxidation (p < 0.05) and protein carbonylation (p < 0.01) was significantly reduced by simultaneous treatment with AEIA or AEEF. The levels of CAT, SOD, GST, GPx, GR, G6PD, GR and GSH were significantly reduced (p < 0.01) in CdCl_2_ intoxicated hepatocytes. Simultaneous treatment of the extracts along with CdCl_2_ significantly reverted (p < 0.05–0.01) the levels of aforementioned antioxidant enzymes and GSH to near-normal status. Excessive regeneration of ROS during Cd-intoxication is associated with the activation of NADPH oxidases. Figure 4a, b confirmed the enhanced (p < 0.01) production of ROS and NADPH in hepatocytes during Cd-intoxication, respectively. However, simultaneous incubation with AEIA or AEEF significantly (p < 0.01) inhibited ROS and NADPH oxidases levels in murine hepatocytes.

**Table 1:** Effect on antioxidant parameters in absence (CdCl_2_) and presence of AEIA (AEIA + CdCl_2_) and AEEF (AEEF + CdCl_2_) in isolated mice hepatocytes

Parameters	Control	CdCl_2_ (30 µM)	AEIA (400 µg/ml) + CdCl_2_ (30 µM)	AEEF (400 µg/ml) + CdCl_2_ (30 µM)
TBARS (µg/g of tissue)	3.65 ± 0.24	6.89 ± 0.54^#^	4.89 ± 0.45*	5.11 ± 0.51*
Protein carbonyl (nmol/mg of protein)	41.75 ± 4.24	76.54 ± 5.33^#^	52.82 ± 4.79**	54.11 ± 3.96**
CAT (U/mg of protein)	234.56 ± 14.33	168.67 ± 10.89^#^	234.56 ± 14.92**	228.92 ± 12.78*
SOD (U/mg of protein)	86.54 ± 4.12	55.54 ± 3.96^#^	75.44 ± 4.06**	72.12 ± 4.35*
GST (µmol/h/mg protein)	0.92 ± 0.11	0.54 ± 0.07^$^	0.88 ± 0.09*	0.83 ± 0.07
GPx (nmol/min/mg of protein)	76.67 ± 4.12	51.78 ± 3.50^#^	71.58 ± 4.21**	67.43 ± 3.98**
GR (nmol/min/mg of protein)	82.01 ± 4.45	54.15 ± 3.92^#^	72.67 ± 4.05*	70.84 ± 4.96*
G6PD (nmol/min/mg of protein)	116.12 ± 5.56	75.60 ± 4.85^#^	104.78 ± 5.11**	101.96 ± 4.76**
GSH (nmol/mg of protein)	5.23 ± 0.47	3.12 ± 0.34^#^	4.81 ± 0.40*	4.67 ± 0.33*

Values are expressed as mean ± SE (n = 3). ^#^Values differ significantly from normal control (p < 0.01). ^$^ Values differ significantly from normal control (p < 0.05). * Values differ significantly from CdCl_2_ control (p < 0.05). ** Values differ significantly from CdCl_2_ control (p < 0.01). CAT unit, ‘U’ is defined as µmoles of H_2_O_2_ consumed per minute. SOD unit, ‘U’ is defined as the µmoles inhibition of NBT reduction per minute.

#### Effect on CdCl_2_ induced alteration of signaling proteins

**Fig. 5:**
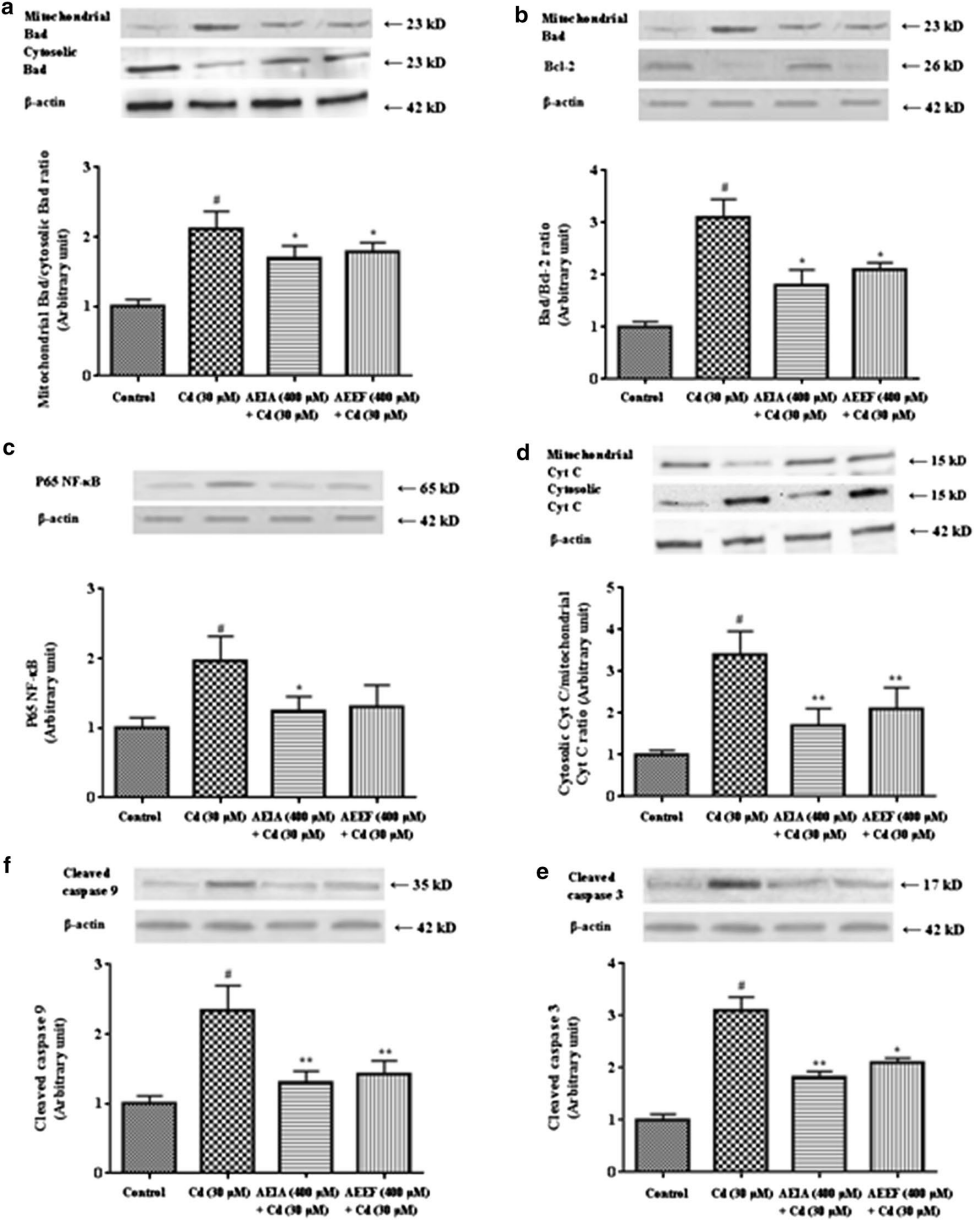
Respective western blot analysis of translocation of Bad to mitochondria (**a**), Mitrocondrial Bad vs Bcl-2 (**b**), P65 NF-κB (**c**), cytosolic vs mitochondrial Cyt C (**d**), cleaved caspase 9 (**e**) and 3 (**f**) in absence (CdCl_2_) and presence of AEIA and AEEF followed by densitometric analysis of the respective protein levels (*bottom*). The normal control band was given an arbitrary value of 1. β-actin was used as a loading protein. Values are expressed as mean ± SE (n = 3). ^#^Values differ significantly (p < 0.01) from normal control. *Values differ significantly (p < 0.05) from toxic control. **Values differ significantly (p < 0.01) from toxic control.

The activation of NADPH oxidases is associated with simultaneous increment in P47 phosphorylation and activation of JNK phophorilation. Later plays an important role in the event of apoptosis. In this study, a significant up-regulation (p < 0.01) of phosphorylated P47 was observed in western blot analysis of Cd-intoxicated murine hepatocytes (Fig. 4c). A significant up-regulation (p < 0.01) of phosphorylated-JNK (both 54 and 46 kD proteins) over total JNK (both 54 and 46 kD proteins) was recorded in CdCl_2_ treated hepatocytes (Fig. 4d). Incubation of hepatocytes with the AEIA or AEEF, however, significantly arrested the phosphorylation of P47 (p < 0.05) and JNK (p < 0.01–0.05) in isolated murine hepatocytes. Apoptosis is regulated by a complex interaction of anti-apoptotic and pro-apoptotic proteins following the activation of caspases. Immunoblotting results suggested that CdCl_2_ up-regulated the translocation of pro-apoptotic Bad in mitochondria from cytoplasm. Figure 5a depicted that an increase in the expression of Bad protein in mitochondria with concomitant down-regulation of Bad expression in cytosol resulting a significant higher (p < 0.01) mitochondrial Bad/cytosolic Bad over control. However, incubation of hepatocytes with AEIA or AEEF significantly (p < 0.05) reverted these alterations near to normalcy. The anti-apoptotic Bcl-2 protein was significantly down regulated following Cd-intoxication, which resulted a significant increase (p < 0.01) in mitochondrial Bad/Bcl-2 ratio (Fig. 5b). However, incubation of hepatocytes with AEIA or AEEF significantly (p < 0.05) reinstated these alterations to near-normal status. To investigate the pro-apoptotic effect of P65 NF-κB in Cd-induced hepatocyte injury, immunoblot analysis of P65 NF-κB showed that CdCl_2_ exposure caused a significant (p < 0.01) increase in the expression of P65 NF-κB (Fig. 5c). However, simultaneous incubation of hepatocytes with AEIA or AEEF significantly (p < 0.05) reduced the expression of P65 NF-κB as compared to toxic control group. The increase in the expressions of cytosolic Cyt C in association with activation of cleaved caspase 3 and 9, demonstrated the association of the mitochondria dependent (intrinsic) apoptotic pathway. In this study, an increase in the expressions of cytosolic Cyt C with simultaneous reduction of mitochondrial Cyt C resulted a significant (p < 0.01) increase in cytosolic Cyt C/mitochondrial Cyt C (Fig. 5d). In this study, the expressions of cleaved fraction of caspase 9 and 3 were up-regulated in the CdCl_2_ intoxicated hepatocytes when compared with controlled hepatocytes (Fig. 5e, f, respectively). It suggested the cleavage of pro-caspase 9 and 3 into respective cleaved fraction (the expressions of pro-caspase expression were not shown). However, treatment of hepatocytes with AEIA or AEEF significantly (p < 0.05-0.01) reinstated the expressions of cleaved caspase 3 and 9, when compared with CdCl_2_ controlled hepatocytes.

**Fig. 6:**
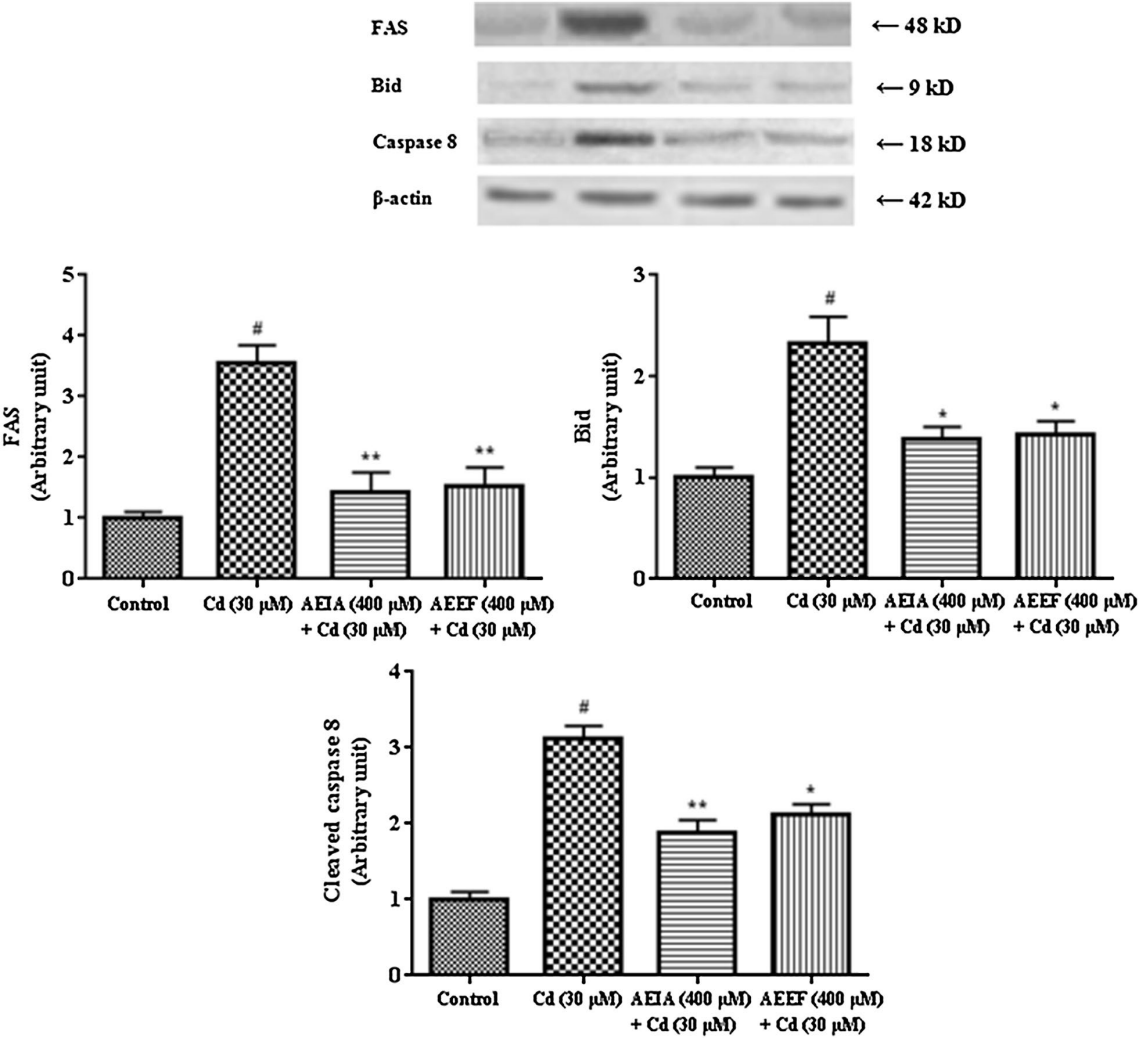
Respective western blot analysis of Fas, Bid, and caspase 8 in absence (CdCl_2_) and presence of AEIA and AEEF followed by densitometric analysis of the respective protein levels (*bottom*). The normal control band was given an arbitrary value of 1. β-actin was used as a loading protein. Values are expressed as mean ± SE (n = 3). ^#^Values differ significantly (p < 0.01) from normal control. *Values differ significantly (p < 0.05) from toxic control. **Values differ significantly (p < 0.01) from toxic control.

Activation of the extrinsic receptor-mediated pathway leads to cause apoptosis via Fas-mediated activation of caspase-8 and Bid. Therefore, expression levels of FAS, Bid and cleaved caspase 8 have been observed via western blot analysis. CdCl_2_ significantly (p < 0.01) up-regulated the expressions of Bid, FAS and cleaved caspase 8 (Fig. 6) in isolated murine hepatocytes. Simultaneous treatment of the cells with AEIA or AEEF, however, significantly reduced the expressions of Fas (p < 0.01), Bid (p < 0.05) and cleaved caspase 8 (p < 0.05-0.01) when compared with CdCl_2_ treated hepatocytes and restore the expressions near-normal status.

### Effect of AEIA and AEEF against CdCl_2_ intoxication in experimental mice

#### Effect on haematological and serum biochemical parameters

The effects of different treatments on haematological and serum biochemical parameters are shown in Table 2. The haematological parameters showed a significant (p < 0.01) reduction in total erythrocyte counts and haemoglobin content in CdCl_2_ treated animals (group II). AEIA and AEEF supplementation significantly reverted the total erythrocyte counts (p < 0.01) and haemoglobin content (p < 0.05). Animals treated with CdCl_2_ showed a significant (p < 0.01) increase in AST, ALT, urea, triglyceride levels, whereas, the levels of HDL cholesterol was found to decrease significantly (p < 0.01). Animals received the combined treatment of extracts and CdCl_2_ showed significant improvements (p < 0.05-0.01) in all the biochemical parameters tested.

**Table 2:** Effect on haematological and serum biochemical parameters in absence (CdCl_2_) and presence of AEIA (AEIA + CdCl_2_) and AEEF (AEEF + CdCl_2_) in mice

Parameters	Group I	Group II	Group III	Group IV
Total erythrocyte count (×10^6/^mm^3^)	6.12 ± 0.31	3.25 ± 0.24^#^	5.05 ± 0.33**	4.78 ± 0.41**
Haemoglobin (g/dl)	8.92 ± 0.85	5.04 ± 0.42^#^	7.45 ± 0.67*	7.34 ± 0.67*
ALT (IU/l)	84.56 ± 5.11	213.34 ± 17.50^#^	132.13 ± 12.92**	148.56 ± 13.11**
AST (IU/l)	52.31 ± 3.24	88.35 ± 4.67^#^	60.89 ± 3.76**	67.33 ± 4.09**
Urea (mg/dl)	25.67 ± 1.89	56.11 ± 2.73^#^	45.14 ± 2.01**	47.92 ± 2.67*
Total cholesterol (mg/dl)	142.13 ± 6.17	198.34 ± 8.22^#^	161.45 ± 7.05**	167.96 ± 7.67*
HDL cholesterol (mg/dl)	58.77 ± 3.22	36.92 ± 2.21^#^	49.87 ± 2.54**	47.21 ± 3.01*
Triglyceride (mg/dl)	125.41 ± 5.12	278.33 ± 11.54^#^	187.85 ± 8.32**	212.50 ± 7.28**

Values are expressed as mean ± SE, for six animals in each group. ^#^Values differ significantly from normal control (p < 0.01). * Values differ significantly from CdCl_2_ control (p < 0.05). ** Values differ significantly from CdCl_2_ control (p < 0.01). Group I: Normal control; Group II: Toxic control; Group III: AEIA (100 mg/kg) + CdCl_2_ (4 mg/kg); Group IV: AEEF (100 mg/kg) + CdCl_2_ (4 mg/kg).

#### Effect of AEIA and AEEF in Cd bioaccumulation

The effects of different treatments in intracellular Cd burden are shown in Table 3. Exposure to CdCl_2_ in mice significantly (p < 0.01) increased the bioaccumulation of Cd in kidney, liver, heart, brain and testes, as compared to untreated animals (group I). The order of intracellular Cd content was kidney > heart > testes > liver > brain. Treatment of AEIA and AEEF significantly (p < 0.05-0.01) prevented intracellular Cd burden in aforementioned tissues, as compared to CdCl_2_ treated animals (group II).

**Table 3:** Effect on intracellular Cd burden in liver, kidney, heart, brain and testes in absence (CdCl_2_) and presence of AEIA (AEIA + CdCl_2_) and AEEF (AEEF + CdCl_2_) in mice

Groups	Cadmium burden (ppm of wet tissue)				
	Liver	Kidney	Heart	Brain	Testes
I	0.07 ± 0.003	0.05 ± 0.007	0.07 ± 0.005	0.04 ± 0.002	0.02 ± 0.003
II	18.56 ± 1.98^#^	24.67 ± 1.88^#^	21.32 ± 2.02^#^	7.23 ± 0.98^#^	1.23 ± 0.13^#^
III	9.42 ± 1.62**	15.04 ± 1.72**	12.34 ± 1.89**	4.12 ± 0.83*	0.85 ± 0.07*
IV	11.21 ± 1.89*	17.33 ± 1.46**	14.02 ± 1.84*	4.41 ± 0.72*	0.91 ± 0.09*

Values are expressed as mean ± SE, for six animals in each group. ^#^Values differ significantly from normal control (p < 0.01). * Values differ significantly from CdCl_2_ control (p < 0.05). ** Values differ significantly from CdCl_2_ control (p < 0.01). Group I: Normal control; Group II: Toxic control; Group III: AEIA (100 mg/kg) + CdCl_2_ (4 mg/kg); Group IV: AEEF (100 mg/kg) + CdCl_2_ (4 mg/kg).

#### Effect on ROS production, lipid peroxidation, protein carbonylation and NADPH oxidase

Cd-intoxication caused enhanced (p < 0.01) accumulation of intercellular ROS in the selected tissues (Table 4). AEIA or AEEF treatment prior to Cd administration significantly (p < 0.05–0.01) prevented the Cd mediated ROS generation and subsequent oxidative stress. Protein carbonylation and lipid peroxidation are chief indicators in oxidative stress-related organ pathophysiology. In this study, the extent of lipid peroxidation has been calculated by evaluating the concentrations of TBARS in kidney, liver, heart, brain and testes of experimental mice (Table 4). CdCl_2_ treated mice (group II) exhibited a significant raise (p < 0.01) in TBARS levels, when compared with normal control group (group I). The maximum extent of lipid peroxidation was found in kidney followed by liver, heart, testes and brain. AEIA and AEEF supplementation, however, significantly prevented that increase in TBARS level in the tissues. The extent of protein carbonylation has also been significantly increased (p < 0.01) in toxic control mice as compared with normal control animals (Table 4). However, AEIA and AEEF treatment significantly attenuated that increase in the levels of protein carbonyl content in selected tissues. Cd significantly (p < 0.01) up-regulated NADPH oxidase in the selected tissues of experimental mice (Table 4). Simultaneous administration of either of AEIA or AEEF significantly (p < 0.05) attenuated NADPH oxidase in the selected organs.

**Table 4:** Effect on Co-enzymes Q in liver, kidney, heart, brain and testes in absence (CdCl_2_) and presence of AEIA (AEIA + CdCl_2_) and AEEF (AEEF + CdCl_2_) in mice

Parameters	Gr	Liver	Kidney	Heart	Brain	Testes
ROS production (nmol DCF/min/mg of protein)	I	28.65 ± 1.67	38.15 ± 2.24	28.15 ± 2.02	33.49 ± 2.23	31.49 ± 1.54
	II	78.56 ± 3.92^#^	86.29 ± 3.48^#^	64.13 ± 3.69^#^	67.09 ± 5.12^#^	71.23 ± 4.28^#^
	III	53.47 ± 2.67**	66.50 ± 4.54**	49.95 ± 3.15*	50.21 ± 3.85*	52.15 ± 3.94**
	IV	59.33 ± 3.45**	71.18 ± 4.12*	51.78 ± 3.88*	52.33 ± 3.08*	56.01 ± 3.42*
Lipid peroxidation (TBARS level in μg/g of tissue)	I	5.11 ± 0.76	8.76 ± 1.01	4.21 ± 0.58	2.34 ± 0.32	3.22 ± 0.32
	II	8.74 ± 0.98^#^	16.15 ± 2.11^#^	8.25 ± 0.58^#^	4.12 ± 0.50^#^	5.58 ± 0.72^#^
	III	5.86 ± 0.54*	10.24 ± 1.54*	5.02 ± 0.45**	2.67 ± 0.24*	3.61 ± 0.42*
	IV	5.94 ± 0.72*	10.58 ± 1.33*	5.21 ± 0.62*	2.86 ± 0.33*	3.78 ± 0.45*
Protein cabonylation (nmol/mg of protein)	I	34.56 ± 1.89	4.87 ± 0.54	2.31 ± 0.22	1.92 ± 0.34	1.02 ± 0.12
	II	72.13 ± 2.67^#^	9.76 ± 1.01^#^	5.02 ± 0.51^#^	3.87 ± 0.56^$^	1.97 ± 0.23^#^
	III	41.32 ± 2.01**	6.65 ± 0.67*	3.28 ± 0.33*	2.24 ± 0.48*	1.32 ± 0.09*
	IV	44.56 ± 2.11**	6.89 ± 0.78*	3.41 ± 0.42*	2.29 ± 0.31*	1.38 ± 0.14*
NADPH oxidase (pmol/min/mg of protein)	I	0.42 ± 0.03	0.56 ± 0.04	0.67 ± 0.03	0.38 ± 0.02	0.32 ± 0.03
	II	3.67 ± 0.55^#^	4.55 ± 0.67^#^	3.91 ± 0.40^#^	2.98 ± 0.33^#^	3.18 ± 0.28^#^
	III	2.45 ± 0.18*	2.85 ± 0.33*	2.44 ± 0.45*	1.85 ± 0.24*	2.24 ± 0.35*
	IV	2.58 ± 0.21*	2.89 ± 0.54*	2.58 ± 0.32*	1.89 ± 0.27*	2.38 ± 0.26
Total coenzyme Q9 (nmol/g of wet tissue)	I	178.67 ± 9.14	148.32 ± 6.33	58.24 ± 3.98	28.67 ± 1.72	98.72 ± 4.92
	II	124.56 ± 7.33^#^	112.56 ± 5.59^#^	39.21 ± 2.21^#^	17.87 ± 1.08^#^	66.67 ± 3.90^#^
	III	164.43 ± 8.39**	144.06 ± 6.18**	53.12 ± 2.92*	24.41 ± 1.45*	84.56 ± 4.04*
	IV	158.25 ± 7.53*	139.33 ± 5.89*	49.32 ± 2.54	24.18 ± 1.52*	81.31 ± 3.78
Total coenzyme Q10 (nmol/g of wet tissue)	I	30.50 ± 2.34	25.67 ± 1.92	27.24 ± 1.98	7.51 ± 0.46	14.21 ± 1.02
	II	18.72 ± 1.78^#^	15.70 ± 1.18^#^	18.67 ± 1.02^#^	4.35 ± 0.22^#^	8.67 ± 0.84^#^
	III	28.33 ± 1.98**	23.65 ± 1.78**	24.89 ± 1.54*	6.12 ± 0.52*	12.33 ± 1.10*
	IV	27.65 ± 2.12*	22.04 ± 1.04*	24.10 ± 1.42	5.78 ± 0.42	11.75 ± 0.78

Values are expressed as mean ± SE, for six animals in each group. ^#^Values differ significantly from normal control (p < 0.01). ^$^ Values differ significantly from normal control (p < 0.01). * Values differ significantly from CdCl_2_ control (p < 0.05). ** Values differ significantly from CdCl_2_ control (p < 0.01). Group I: Normal control; Group II: Toxic control; Group III: AEIA (100 mg/kg) + CdCl_2_ (4 mg/kg); Group IV: AEEF (100 mg/kg) + CdCl_2_ (4 mg/kg).

#### Effect on co-enzymes Q

The effect of various treatments on mitochondrial co-enzymes Q (Q_9_ and Q_10_) in kidney, liver, heart, brain and testes has been estimated (Table 4). Total co-enzyme Q_9_ and Q_10_ levels in the selected tissues were significantly (p < 0.01) reduced in CdCl_2_ treated animals (group II). Treatment with AEIA significantly elevated Q_9_ and Q_10_ levels in liver (p < 0.01), kidney (p < 0.01) heart (p < 0.05), brain (p < 0.05) and testes (p < 0.05). On other hand, AEEF treatment significantly elevated Q_9_ levels in hepatic (p < 0.05), renal (p < 0.05) and cerebral (p < 0.05) tissues and Q_10_ levels in hepatic (p < 0.05) and renal (p < 0.05) of experimental mice. However, no significant alteration of co-enzyme Q_9_ levels in heart and testes and Q_10_ levels in heart, brain and testes was observed in AEEF + CdCl_2_ treated group when compared with toxic control (group II).

#### Effect on antioxidant enzymes and reduced glutathion

The antioxidant enzymes protect biological macromolecules from oxidative damage in oxidative stress. Therefore, the activities of antioxidant enzymes (CAT, SOD, GST, GPx, GR and G6PD) and other cellular metabolites (GSH) have been estimated in this study (Table 5). CAT levels were significantly decreased in liver (p < 0.01), kidney (p < 0.01), heart (p < 0.01), brain (p < 0.01) and testes (p < 0.05) of CdCl_2_ treated mice (group II) as compared with normal mice (group I). However, treatment with AEIA significantly (p < 0.05-0.01) increased CAT activities in aforementioned tissues, while AEEF improved CAT levels in hepatic (p < 0.05), renal (p < 0.05) and cardiac (p < 0.05) tissues significantly. In the study of SOD activities, a significant (p < 0.05-0.01) depletion of SOD level was observed in the selected tissues of CdCl_2_ intoxicated mice (group II). Treatment with AEIA significantly improved hepatic (p < 0.01), renal (p < 0.01), cardiac (p < 0.05), cerebral (p < 0.05) and testicular SOD (p < 0.05) levels. However, AEEF produced positive effect in hepatic (p < 0.01), renal (p < 0.05) and cardiac (p < 0.05) tissues. In the study of reduced glutathione regulated enzymes namely, GST, GPx, G6PD and GR, a significant (p < 0.01) reduction in the levels of aforementioned enzymes was observed in CdCl_2_ control mice (group II). The treatment of AEIA significantly (p < 0.05-0.01) increased the levels of GST and GPx in all the selected tissues. However, AEIA treatment significantly improved GR levels in liver (p < 0.05), kidney (p < 0.01), heart (p < 0.01) and testes (p < 0.05) and G6PD levels in liver (p < 0.05), kidney (p < 0.01) and heart (p < 0.05) of experimental mice (group III). In another side, treatment of AEEF significantly (p < 0.05) increase the levels of GST and GPx in hepatic, renal, cardiac and testicular tissues. However, AEEF treatment significantly improve GR levels in kidney (p < 0.05) and heart (p < 0.05), and G6PD levels in liver (p < 0.05) and kidney (p < 0.05) of experimental mice (group IV). CdCl_2_ intoxication significantly (p < 0.01) reduced GSH levels in the selected tissues of experimental mice (group II). However, AEIA treatment significantly improved GSH levels in liver (p < 0.05), kidney (p < 0.01), heart (p < 0.05), brain (p < 0.05) and testes (p < 0.05) of experimental mice (group III) when compared with toxic control animal (group II). On other hand, AEEF treatment improved GSH levels in liver, kidney and testes of experimental mice (group IV) when compared with toxic control animal (group II).

**Table 5:** Effect on antioxidant enzymes and GSH levels in liver, kidney, heart, brain and testes in absence (CdCl_2_) and presence of AEIA (AEIA + CdCl_2_) and AEEF (AEEF + CdCl_2_) in mice

Parameters	Group	Liver	Kidney	Heart	Brain	Testes
CAT (U/mg of protein)	I	312.92 ± 27.65	248.22 ± 21.24	313.24 ± 21.33	94.12 ± 6.67	41.25 ± 3.87
	II	178.43 ± 16.86^#^	128.30 ± 14.04^#^	192.56 ± 17.04^#^	46.45 ± 3.92^#^	25.54 ± 3.01^$^
	III	279.88 ± 25.46**	221.33 ± 23.78**	267.54 ± 16.92*	69.75 ± 6.78*	38.76 ± 2.98*
	IV	264.20 ± 27.11*	214.34 ± 17.67*	261.41 ± 18.00*	67.22 ± 5.98	35.43 ± 3.93
SOD (U/mg of protein)	I	82.17 ± 7.21	78.02 ± 6.89	134.69 ± 10.17	94.52 ± 6.25	119.42 ± 8.13
	II	45.83 ± 4.34^#^	42.34 ± 4.71^#^	78.35 ± 8.92^#^	52.24 ± 5.08^#^	78.34 ± 7.78^$^
	III	76.51 ± 6.94**	72.86 ± 6.94**	119.85 ± 11.01*	78.92 ± 7.38*	111.85 ± 9.25*
	IV	75.33 ± 6.23**	69.87 ± 7.18*	119.61 ± 9.87*	73.05 ± 7.01	103.61 ± 9.96
GST (µmol/h/mg protein)	I	1.28 ± 0.18	1.34 ± 0.11	0.64 ± 0.06	1.75 ± 0.15	4.51 ± 0.36
	II	0.59 ± 0.07^#^	0.68 ± 0.05^#^	0.42 ± 0.03^#^	1.24 ± 0.09^$^	2.45 ± 0.21^#^
	III	1.16 ± 0.14*	1.21 ± 0.10**	0.61 ± 0.04*	1.67 ± 0.09*	3.91 ± 0.40*
	IV	1.14 ± 0.09*	1.20 ± 0.08*	0.59 ± 0.05*	1.65 ± 0.12	3.78 ± 0.33*
GPx (nmol/min/mg protein)	I	123.85 ± 6.67	43.62 ± 3.78	312.15 ± 16.33	165.43 ± 8.94	162.05 ± 7.65
	II	78.50 ± 4.42^#^	26.95 ± 2.21^#^	206.96 ± 12.28^#^	121.35 ± 6.67^#^	112.33 ± 6.10^#^
	III	108.50 ± 5.33**	39.28 ± 2.89*	278.36 ± 17.82*	155.85 ± 8.12*	148.76 ± 6.05**
	IV	102.05 ± 5.12*	38.47 ± 3.01*	272.84 ± 14.95*	148.33 ± 7.33	142.50 ± 7.02*
GR (nmol/min/mg protein)	I	88.85 ± 6.33	34.51 ± 2.87	82.17 ± 4.01	48.12 ± 4.49	85.10 ± 4.54
	II	61.78 ± 4.18^#^	19.33 ± 2.02^#^	42.76 ± 2.19^#^	28.94 ± 2.86^#^	52.95 ± 4.20^#^
	III	84.33 ± 8.02*	31.24 ± 2.49**	63.21 ± 3.77**	40.70 ± 3.55	69.43 ± 3.36*
	IV	77.36 ± 5.79	29.72 ± 1.92*	58.76 ± 3.54*	38.29 ± 3.02	63.21 ± 4.47
G6PD (nmol/min/mg protein)	I	104.32 ± 9.12	86.74 ± 7.12	98.32 ± 5.14	34.68 ± 3.06	54.25 ± 4.72
	II	54.31 ± 5.76^#^	45.17 ± 3.22^#^	69.28 ± 4.21^#^	19.43 ± 2.33^#^	34.56 ± 2.89^#^
	III	86.08 ± 7.11*	72.16 ± 5.15**	89.67 ± 4.75*	26.54 ± 1.76	46.29 ± 3.16
	IV	85.33 ± 6.58*	67.21 ± 4.99*	86.23 ± 4.77	25.33 ± 1.58	45.02 ± 3.04
GSH (nmol/mg protein)	I	24.21 ± 2.54	16.54 ± 1.01	19.51 ± 2.01	27.12 ± 2.67	17.65 ± 1.87
	II	13.21 ± 1.69^#^	8.33 ± 0.82^#^	9.34 ± 1.54^#^	13.25 ± 1.48^#^	9.67 ± 1.00^#^
	III	21.88 ± 2.05*	13.45 ± 1.15**	15.43 ± 1.67*	18.24 ± 1.92*	15.67 ± 1.24*
	IV	21.35 ± 1.90*	12.67 ± 0.98*	14.45 ± 1.48	19.12 ± 1.78	15.02 ± 1.61*

Values are expressed as mean ± SE, for six animals in each group. ^#^Values differ significantly from normal control (p < 0.01). ^$^ Values differ significantly from normal control (p < 0.05). * Values differ significantly from CdCl_2_ control (p < 0.05). ** Values differ significantly from CdCl_2_ control (p < 0.01). Group I: Normal control; Group II: Toxic control; Group III: AEIA (100 mg/kg) + CdCl_2_ (4 mg/kg); Group IV: AEEF (100 mg/kg) + CdCl_2_ (4 mg/kg).

#### Effect on histology of organs

**Fig. 7:**
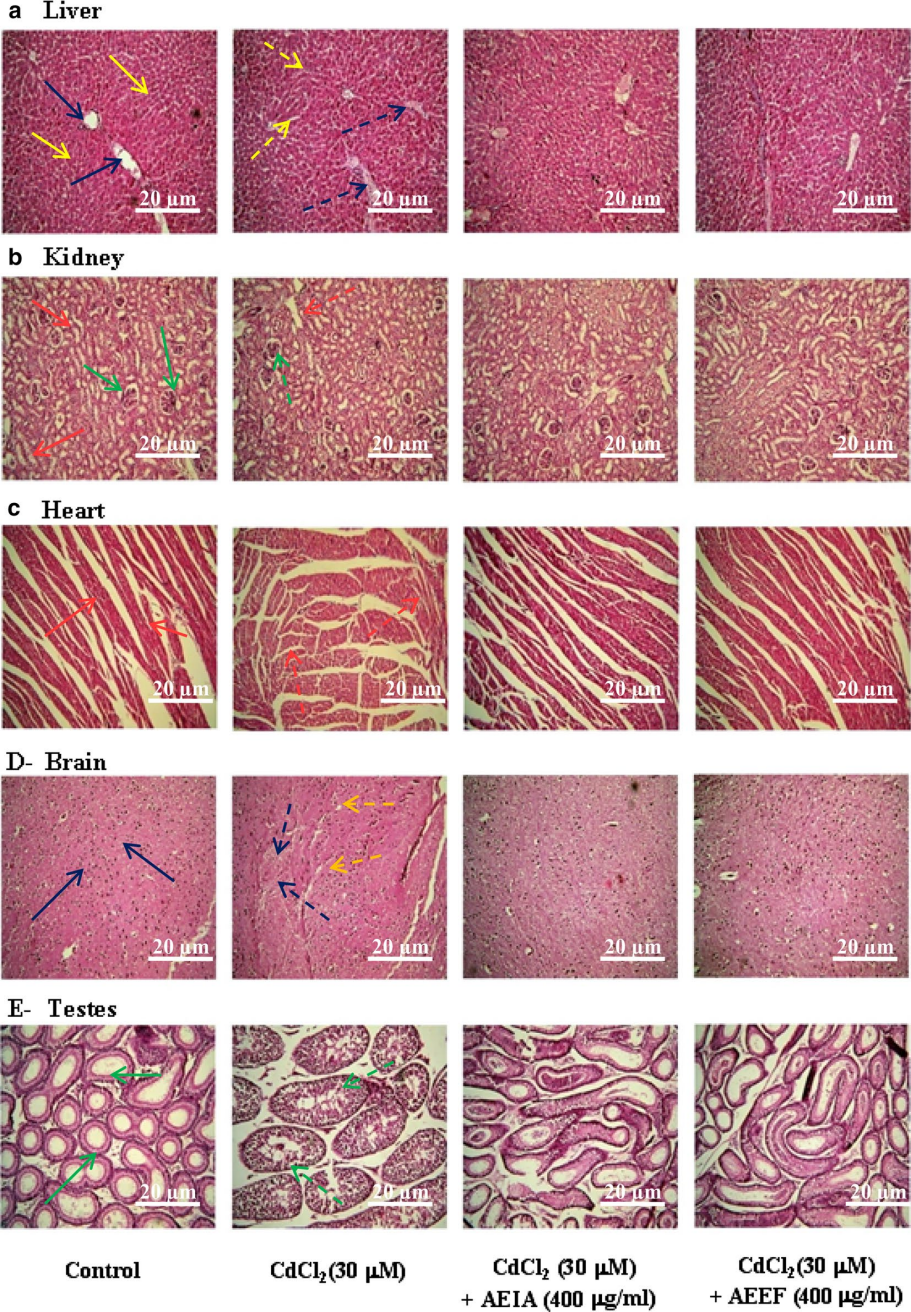
Histological sections of different tested organs of experimental mice in absence (CdCl_2_) and presence of AEIA and AEEF. Untreated mice were kept as control to compare the structural changes caused by CdCl_2_. **a** Histogram of liver sections; *blue* and *yellow* *solid* *arrows* represent normal portal vein and hepatocytes, respectively; *dotted arrows* represent the CdCl_2_ mediated structural changes of portal veins (*blue dotted*) and hepatocytes (*yellow dotted*). **b** Histogram of kidney sections; *green* and *red* *solid* *arrows* represent normal glomerular structures and renal tubules, respectively; *dotted* *arrows* represent the CdCl_2_ mediated glomerular hypercellularity (*green dotted*) and swelling of renal tubules (*red dotted*). **c** Histogram of heart sections; *red solid arrows* indicated normal radiating pattern of cardiac muscle; *red dotted arrows* showed extensive degeneration in cardiac muscle during CdCl_2_-intoxication. **d** Histogram of brain sections; *blue arrows* represent normal cyto-architecture of brain; *dotted arrows* represent the CdCl_2_ mediated development of vacuolated area of degenerated tissues (*blue dotted*) and diffused edema (*yellow dotted*). **e** Histogram of testes sections; *green arrows* represent normal seminiferous tubules; *green dotted arrows* represent CdCl_2_ mediated marked degeneration in the seminiferous tubules in testes section.

The histological assessments of different organs viz. liver, kidney, heart, brain and testes have been depicted in Fig. 7a–e respectively. The results revealed that the liver of CdCl_2_ treated mice exhibited diffused portal tract and hepatocyte focal damage. The histological examination of the kidney tissues of the toxic control mice showed glomerular hypercellularity and swelling of renal tubules when compared with normal control animals. Cd-intoxication caused extensive degeneration in cardiac muscle following disorganization of normal radiation pattern. The histopathological examination of brain of Cd-intoxicated mice exhibited vacuolated area degenerated tissues and diffused edema as compared to normal control mice. Severe degeneration of seminiferous tubules was observed in testes of Cd-treated mice with fractional loss of spermatogenic cells. However, treatment with AEIA or AEEF restored the cellular architectures of aforementioned organs near to normalcy i.e. comparable to that of organ sections of normal control group.

## Discussion

Cd possesses pro-oxidant catalytic activity, which leads to oxidative damage of membrane lipids by excessive free radicals-generation followed by inhibition of the intracellular antioxidant defence system within the system [36]. Oxidative stress is known to alter the expressions of genes encoding several redox sensitive transcription proteins, which is important event in initiation of apoptosis in critical organs by both the intrinsic and extrinsic pathways. Present study describes the protective role of aqueous extract of two edible plants, *I. aquatica* and *E. fluctuans,* against Cd toxicity. Both in vitro and in vivo models were employed to evaluate the protective role of test extracts against Cd-intoxication. The special emphasis was given on signaling pathways and evaluation of the mechanism of protection. Finally, the activity has been correlated with the phytochemical present within the extracts.

In this study, CdCl_2_ treatment caused a significant elevation of oxidative stress in murine hepatocyte followed by apoptosis. Earlier studies revealed that oxidative free radicals participates crucial role in Cd mediated cellular damage and responsible to exert oxidative injury to the cellular macromolecules like proteins, lipids and nucleic acids [37]. In this study, CdCl_2_ exerted a significant reduction in number of viable cells of murine hepatocytes. The flow cytometric analysis confirmed that apoptosis is the principle pathway of cellular damage during CdCl_2_ intoxication. Hoechst staining of Cd-intoxicated hepatocytes confirmed the observation of cell viability assay and induction of apoptosis. Moreover, a significantly less number of visible nucleus coupled with distinct pattern of visible nuclei were noticed in Hoechst staining of CdCl_2_-intoxicated hepatocytes. However, contemporaneous incubation of hepatocytes with either of extracts could inhibit the apoptosis.

The generation of ROS is thought to be the principle mechanism of Cd toxicity. In this study, Cd-mediated oxidative stress could be correlated to the increase in the levels of lipid peroxidation and protein carbonylation. However, extract treatment could prevent the Cd-induced augmented lipid peroxidation and protein carbonylation probably by reducing and/or detoxifying several reactive intermediates viz. hypochlorous acid, nitric oxide, hydrogen peroxide and hydroxyl radical etc. [5]. Presence of dietary antioxidants like phenolic compounds, flavonoids and ascorbic acid would be responsible for reduction of augmented oxidative stress caused by CdCl_2_.

The generation of ROS during in presense of Cd is associated with the activation of NADPH oxidase system, which is coupled with an increase in phospho-p47 [38]. NADPH oxidase uses electrons from intracellular NADPH to generate superoxide from molecular oxygen [39]. The NADPH oxidase participates in key cellular processes, associated with cell-signalling, cell-proliferation and apoptosis [40]. NADPH oxidase plays an important role in apoptosis via up-regulation phospho-JNK [20].

Mitochondrial antioxidant enzyme (CAT, SOD, GST, GPx, G6PD, GR etc.) system serves as a line of cellular defense against oxidative stress [41–43]. On other hand, thiol-based antioxidant (GSH) contributes in cellular defense against oxidative damage caused by free radicals. Depletion of aforementioned cellular redox markers during Cd-intoxication may be due to the thiol affinity of Cd and/or impairment of electron transfer process [1, 5]. The extracts may exert their effect by diverse mechanisms, mainly by quenching, addition and/or recombination of free radical and electron transfer.

Oxidative stress is a key player in mitochondrial dysfunction which is an important early event of apoptotic pathway. A number of apoptosis-inducer proteins are up-regulated in presence of oxidative radicals. Apoptotic pathways are executed by some pro-apoptotic (Bad) and anti-apoptotic (Bcl-2) proteins and caspases. Bcl-2 family members regulate apoptosis via intrinsic pathway following migration of cytochrome c from mitochondria to cytosol [5]. Translocation of Bad, a pro-apoptotic protein, from cytosol to mitochondria demonstrated the initiation of apoptotic event. BH-3 family members (Bad, Bid, Bim, etc.) are well-known pro-apoptotic proteins and can change the pro-apoptotic processes by inhibiting the transcription levels of anti-apoptotic proteins [2]. Activation of the extrinsic receptor-mediated pathway induces apoptosis by Fas-mediated activation of Bid and cleavage of pro-caspase 8 to its cleaved fraction [2]. In the present study, immunoblot analysis confirmed the involvement of both intrinsic and extrinsic pathways of apoptosis following Cd exposure. One potential target of oxidative free radicals is P-65 NF-κB. Activation of P-65 NF-κB could participate in various toxicological responses via. inflammation as well induction of apoptosis [44]. However, the extracts could significantly interrupt the apoptotic event may be by inhibiting ROS.

To validate the in vitro protective effect of AEIA or AEEF, in vivo experimentation was performed. The LD_50_ value of CdCl_2_ has been found to be 4 mg/kg in experimental mice. Therefore, 4 mg/kg dose has been chosen for the subsequent experiments. This value is nearly equivalent to ~80 µg/day (for 20 g mice), which is interestingly comparable to the exposure at the highly Cd polluted locations. The amounts of daily Cd ingestion with food in most countries are ranging between 10 and 20 µg/day [45]. Smoking also potentiates the daily consumption of Cd. Therefore, selected dose could be justified through the aforementioned points. The effects of test extracts were examined by evaluating haematological and serum biochemical parameters. Kidney, heart, liver, brain and testes are considered to be the potential targets of Cd toxicity [5]. Therefore, the biochemical and redox markers were analysed for aforementioned tissues along with histological investigation to observe the effect of test extracts in vivo.

In vivo study revealed that Cd was accumulated mostly in kidney and heart followed by liver, brain and minimum quantity in testes. Simultaneous administration of extracts and CdCl_2_ significantly lowered the Cd burden in the selected tissues of experimental mice. The experimental outcome indicated that both the extracts could promote the clearance of intercellular Cd. The phytochemicals present within the extracts might form soluble chelate with Cd and potentiate its clearance from the tissues. Earlier studies revealed the metal chelating activity of flavonoids and saponins [5, 42, 46–49]. Therefore, the reduction of Cd burden within the tissue may be due to presence of significant quantity of flavonoids and saponins in AEIA and AEEF.

The serum biochemical and haematological parameters serve as the earliest indicators of the abnormalities within the system [5]. Considerable reduction in total erythrocyte count and haemoglobin content was observed in CdCl_2_-intoxicated mice, which is in accordance to earlier reports [5, 50]. Increased levels of ALT and AST in serum indicated the membrane leakage during cellular damages in kidneys and livers during Cd-intoxication [5]. Administration of the extracts along with toxin, however, could significantly protect the cellular damage indicated by the reduced levels of aforementioned enzymes in serum. Kidney remained a major target in Cd toxicity. An increase in the level of serum urea may be associated by the protein catabolism due to nephrotoxic effect of Cd. Elevated levels of serum lipids is an indication of increased lipogenesis and/or decreased clearance of lipoproteins during Cd-intoxication. Treatment of AEIA or AEEF could signify the protective role of extract against Cd toxicity.

In search of effect of AEIA and AEEF on tissue antioxidant markers in vivo, it was found that administration of either of extracts along with CdCl_2_ could significantly attenuate the oxidative challenge caused by Cd. The in vivo results supported the in vitro findings. Mitochondrial co-enzymes Q (Q9 and Q10) play an important role as electron carriers. Co-enzymes act as antioxidants by scavenging free radicals and inhibiting lipid peroxidation [51]. The decreased level of coenzyme Q9 and Q10 suggested the oxidative challenge caused by Cd, which was significantly improved by the extracts’ treatment. The extracts might exert this effect through promoting Cd clearance from the tested organs.

Finally, histological assessments revealed that Cd caused abnormal histological changes in the tissues. However, pre-treatment with the extracts could attenuate these histological changes and restore cyto-architecture near-normal status.

## Conclusion

**Fig. 8:**
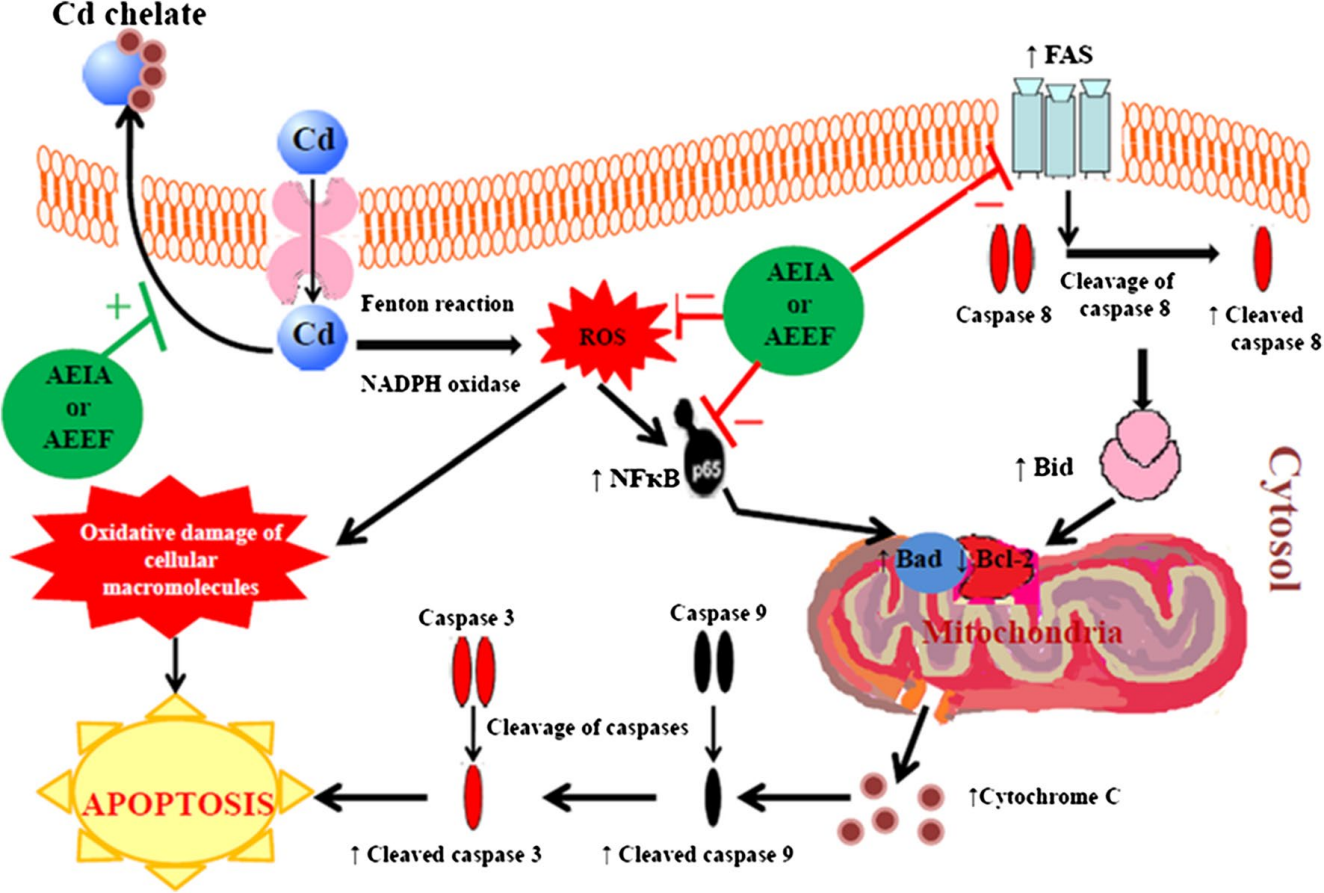
Schematic presentation of probable protective mechanism of AEIA and AEIA against Cd toxicity. The *black arrows* indicate the sequential events involved in Cd pathogenesis. The *red colour lines* (−) indicates inhibitory effect on the events involved in Cd toxicity pathway, while, *green colour line* (+) denotes promoting the process of Cd clearance from the cell.

Existing literature revealed that, Cd accumulation causes redox imbalance which is a principle mechanism in Cd-toxicity (Fig. 8). Therefore, inhibition of Cd bioaccumulation i.e. promoting Cd clearance and/or counteracting with oxidative stress would be the major therapeutic target. In present investigation, it was found that the administration of the aqueous extracts of the edible aerial parts of *I. aquatica* and *E. fluctuans*, individually, attenuated toxic manifestations caused by CdCl_2_. Experimental data showed that excess of oxidative free radicals after Cd exposure caused oxidative tissue damage and initiation of apoptosis by intrinsic as well as extrinsic pathways. The results of the present study suggested that both the extracts offered protection against Cd-induced toxicity by counteracting oxidative stress and ROS mediated apoptosis. The extracts would offer the overall protective effect via counteracting with Cd mediated oxidative stress and/or promoting the elimination of Cd by chelating (Fig. 8). Phytochemical studies revealed presence of flavonoids, phenolic compounds and ascorbic acid in the test extracts. Substantial quantities of aforementioned dietary antioxidants [52, 53] could contribute in overall protection against Cd-intoxication. In conclusion, dietary antioxidants would serve as a protective measure against heavy metals toxicity and would serve as potential to develop new therapy in future without producing countable side effect.
